# Simplified Assessment of Erectile Dysfunction in Adult Men With Sickle Cell Disease: A Cross-Sectional Validation Study

**DOI:** 10.7759/cureus.99066

**Published:** 2025-12-12

**Authors:** Lucas Neves de Oliveira, Jair Bomfim Santos, Anna Paloma Martins Rocha Ribeiro, Caroline Silva, Ricardo Brianezi Tiraboschi, Jose Bessa Junior

**Affiliations:** 1 Department of Public Health, State University of Feira de Santana, Feira de Santana, BRA; 2 Division of Urology, State University of Feira de Santana, Feira de Santana, BRA

**Keywords:** diagnostic test accuracy, erectile dysfunction, erection hardness score, priapism, sickle cell disease

## Abstract

Objective: To estimate the prevalence of erectile dysfunction (ED) and assess the diagnostic performance of simplified tools - the Erection Hardness Score (EHS), the Massachusetts Male Aging Study (MMAS) single-item scale, and a visual analog “Erectometer” - against the International Index of Erectile Function-5 (IIEF-5) among adult men with sickle cell disease (SCD).

Methods: Cross-sectional validation study including 33 adult men with confirmed SCD followed at a regional reference center in Bahia, Brazil (July-December 2021). Sociodemographic, clinical, and laboratory data (total and free testosterone, luteinising hormone (LH), follicle stimulating hormone (FSH), estradiol, ferritin) were collected. Erectile function was assessed using IIEF-5 (reference standard), EHS, MMAS single-item, and the Erectometer. Receiver operating characteristic (ROC) curves and area under the curve (AUC) quantified diagnostic accuracy.

Results: Median age was 35 [IQR 28-48] years; 33% reported prior ischemic priapism. By IIEF-5, 15/33 (45.5%) had moderate-severe ED (≤16). AUCs against IIEF-5 were 0.85 for EHS, 0.82 for MMAS, and 0.90 for the Erectometer (all p<0.001), with sensitivities ≥ 81% and specificities ≥ 79%.

Conclusion: ED is frequent in Brazilian adults with SCD. Brief instruments (EHS, MMAS single-item, Erectometer) show excellent agreement with IIEF-5 and appear suitable for routine screening, potentially enabling earlier multidisciplinary care.

## Introduction

Sickle cell disease (SCD) is among the most prevalent monogenic disorders worldwide, driven by a single β-globin mutation that promotes hemoglobin S polymerization, vaso-occlusion, and chronic hemolysis [1-4]. Cumulative organ damage is typical, and men frequently face urogenital complications, chiefly ischemic priapism and erectile dysfunction (ED), that carry durable effects on quality of life [5-9]. In Brazil, SCD has a marked geographic gradient; Bahia is recognized for particularly high burden, with newborn screening data and national reviews documenting some of the country’s highest incidences and sickle-trait frequencies, making the region uniquely relevant for research and service planning [10-13].

ED in SCD is multifactorial. Recurrent hypoxia-reperfusion, oxidative stress, and hemolysis reduce nitric oxide bioavailability and disrupt cavernosal smooth muscle homeostasis; over time, stuttering and full ischemic priapism episodes can lead to corporal fibrosis and veno-occlusive dysfunction [14-18]. Hypogonadism - whether primary or functional - appears to contribute in some men, potentially exacerbated by iron overload and chronic inflammation [19-21]. These mechanisms dovetail with the epidemiology: across settings, men with SCD report higher rates of sexual dysfunction than non-SCD peers, and a history of priapism consistently tracks with worse erectile outcomes [9,22-24]. Despite this burden, sexual health is seldom addressed systematically in routine SCD care.

Comprehensive questionnaires such as the International Index of Erectile Function (IIEF) are seldom used in busy clinics and may be challenging to implement in low-literacy settings. Brief, validated instruments, including IIEF-5, the Erection Hardness Score (EHS), and single-item screens, offer a pragmatic alternative that better fits short consultations while preserving diagnostic accuracy [25]. However, data on the performance of such simplified tools, particularly in men with SCD in Brazil, remain scarce.

Against this backdrop, we aimed to estimate the prevalence of ED and to evaluate the diagnostic performance of simplified screening instruments compared with IIEF-5 among adult men with SCD receiving routine care in Bahia. The present study was designed with three explicit objectives: (1) to estimate the prevalence of erectile dysfunction among adult men with sickle cell disease; (2) to evaluate the diagnostic performance of simplified screening instruments; and (3) to validate these simplified tools against the IIEF-5 as the reference standard.

## Materials and methods

Study design and setting

We conducted a cross-sectional diagnostic validation study between July and December 2021 at the Sickle Cell Reference Center in Feira de Santana, Bahia, Brazil.

The study protocol was approved by the Research Ethics Committee of Universidade Estadual de Feira de Santana (CAAE 38721120.8.0000.0053, position statement 4.484.589), and all participants provided written informed consent before inclusion.

Participants

**Figure 1:**
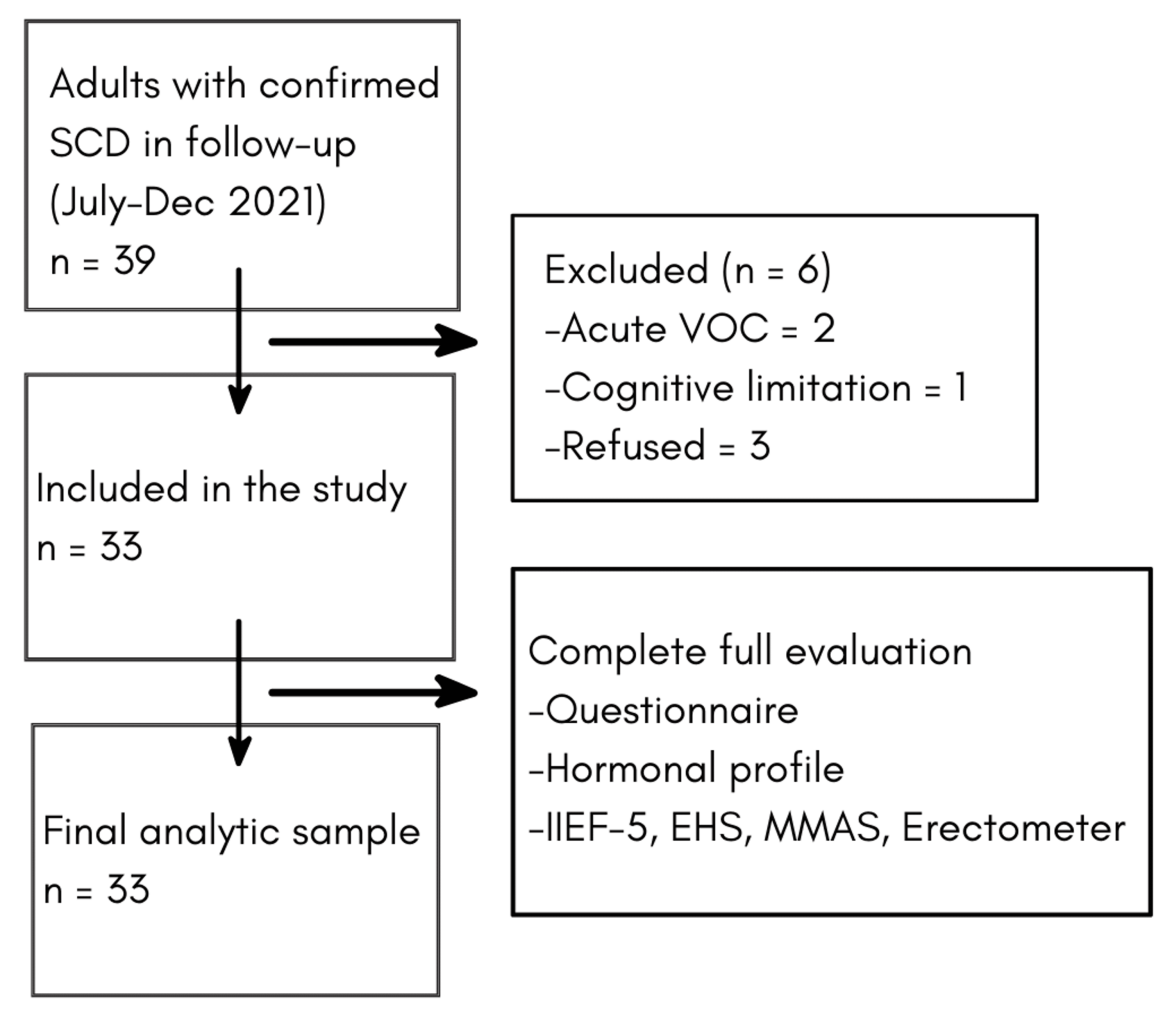
Cohort Flow Chart SCD: Sickle Cell Disease. VOC: Vaso-Occlusive Crisis. IIEF-5: International Index of Erectile Function-5. EHS: Erection Hardness Score. MMAS: Massachusetts Male Aging Study.

We included men aged ≥18 years with confirmed SCD (HbSS, HbSC, or HbSβ) who were regularly followed at the center. Exclusion criteria were cognitive or communication limitations precluding interview, acute vaso-occlusive crisis at the time of assessment, or refusal to participate (Figure 1). Consecutive eligible patients attending routine visits during the study period were invited.

Procedures and measures

Trained staff administered a standardized questionnaire to obtain sociodemographic and clinical data. It collected fasting morning blood samples (07:00-09:00) for routine laboratory parameters, a hormonal profile (including total and free testosterone), and ferritin.

**Figure 2:**
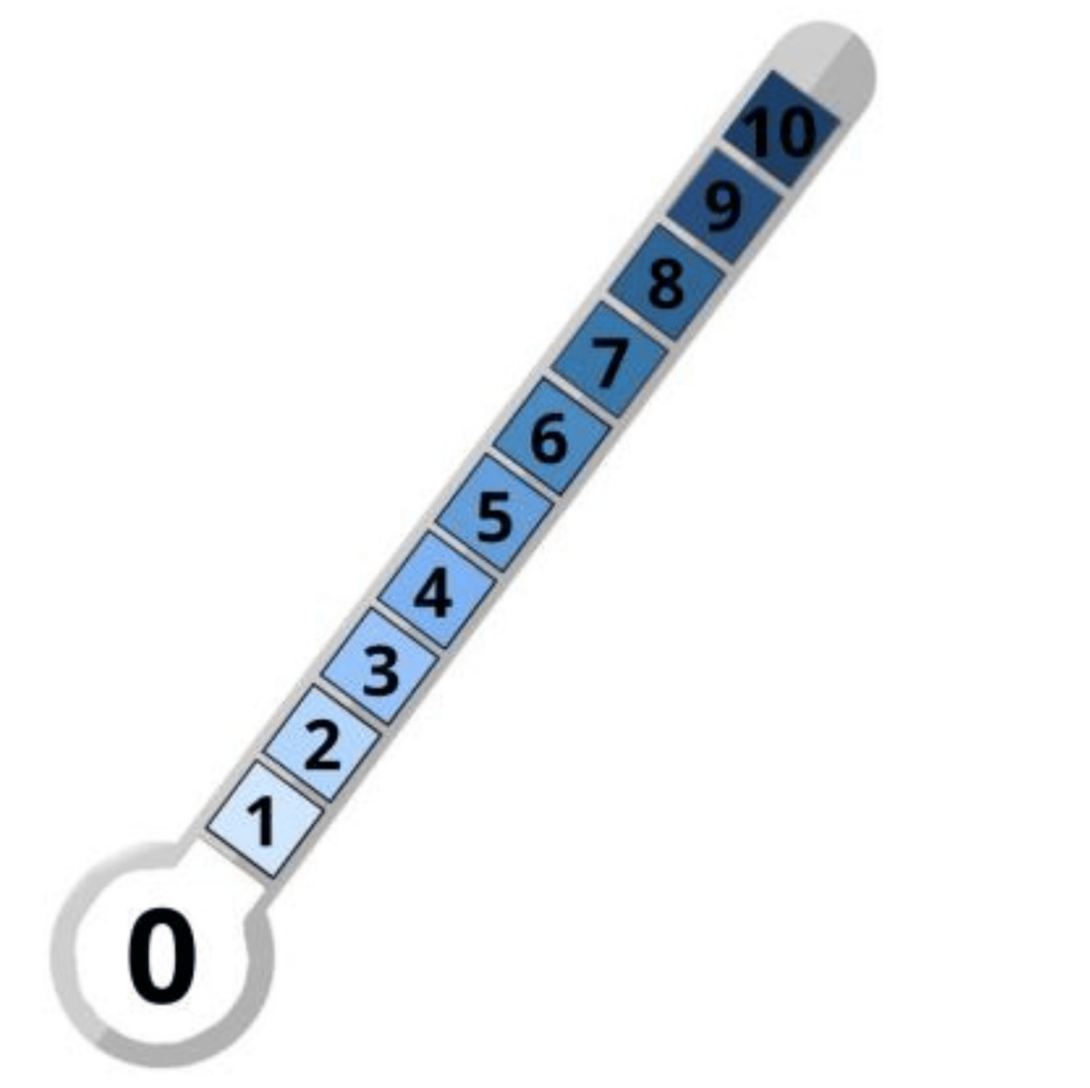
Erectometer - a Visual Analogic Scale (VAS) for assessing erection quality Source: Author's own work

Erectile function simplified instruments were the IIEF-5 (reference): five items, total 5-25; ≤21 indicates ED; ≤16 defines moderate-severe ED [23,26]; the EHS: single-item rigidity scale (0-4), with <3 Indicating insufficient rigidity for penetration [24,27]; the MMAS single-item: frequency of adequate erections (1=never to 4=always) [26,28]; and the Erectometer (Figure 2): 1-10 Visual Analogic Scale (VAS) for assessing self-rating erection quality [29,30].

Participants were consecutively recruited during routine follow-up visits, and no eligible individuals were excluded after recruitment. All instruments used (IIEF-5, EHS, MMAS-style item, Erectometer) are validated measures or previously described tools with documented psychometric properties. Hormonal assays were performed using standardized laboratory protocols on calibrated platforms. Statistical models were fully specified with predefined covariates, and analyses were performed using GraphPad Prism 8.3.0 (GraphPad Software, San Diego, CA, USA).

Statistical analysis

Continuous variables are reported as median [interquartile range, IQR] or mean ± standard deviation (SD), and categorical variables as absolute and relative frequencies. Diagnostic performance of simplified tools versus IIEF-5 (moderate-severe ED defined as ≤16 points) was assessed using receiver operating characteristic (ROC) curves and area under the curve (AUC), with sensitivity and specificity estimated at prespecified cutoffs for each instrument. Correlations between instruments were explored using Spearman rank coefficients. All tests were two-tailed, with statistical significance set at p<0.05.

## Results

Thirty-three men were assessed; median age 35 years [IQR 28-48]. Hydroxyurea use was reported in 73% of cases. Eleven participants (33%) had a history of ischemic priapism. Mean total testosterone was 383 ± 122 ng/dL; mean free testosterone was 10.4 ± 3.1 pg/mL, and median ferritin was 531 [IQR 388-712] ng/mL.

Prevalence and severity of ED

By IIEF-5, 15/33 men (45.5%) met criteria for moderate-severe ED (score ≤16), while 18/33 (54.5%) had mild or no dysfunction (score ≥17). Among those with ED, 9/33 (27.3%) were classified as moderate and 6/33 (18.2%) as severe.

Diagnostic performance of simplified tools

**Figure 3:**
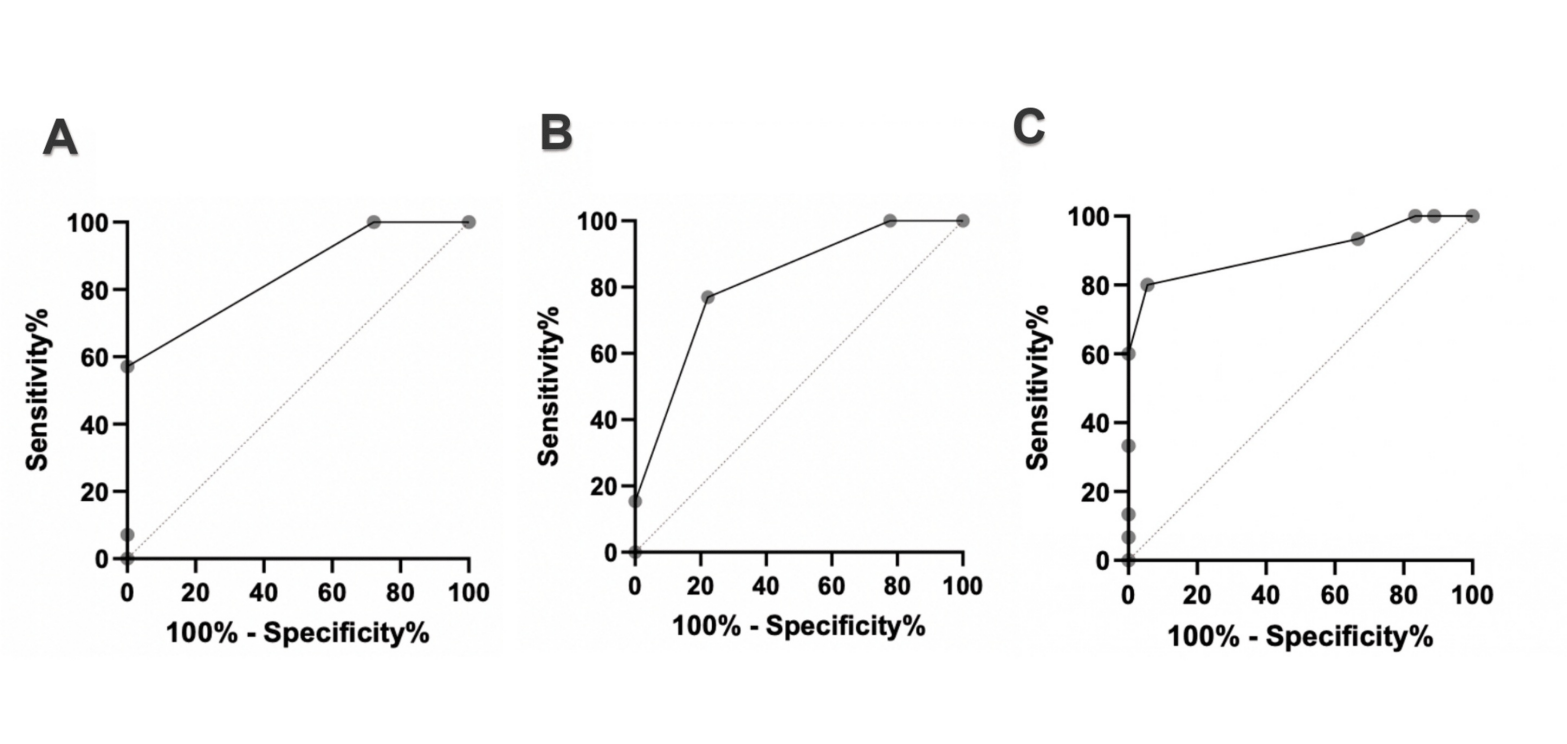
ROC curves of simplified instruments for erectile dysfunction assessment in men with sickle cell disease: (A) EHS, (B) MMAS, (C) Erectometer. ROC: Receiver Operating Characteristic. EHS: Erection Hardness Score. MMAS: Massachusetts Male Aging Study.

In the ROC analyses using the IIEF-5 as the reference standard (Figure 3), all simplified instruments demonstrated good discriminatory ability for identifying moderate-to-severe erectile dysfunction. The EHS showed an AUC of 0.85, with a sensitivity of 88% and specificity of 83% (p<0.0001). The MMAS single-item also performed well, yielding an AUC of 0.82, with sensitivity and specificity of 81% and 79%, respectively (p=0.0007). Among the evaluated tools, the Erectometer showed the highest diagnostic accuracy, with an AUC of 0.90, sensitivity of 91%, and specificity of 87% (p < 0.0001).

## Discussion

Our findings highlight an aspect of sickle-cell care often considered secondary or postponed, despite its crucial role in well-being. In this relatively young group (median age 35 years), nearly half of the men met criteria for moderate to severe ED, consistent with previous reports from specialized centers and community samples showing increased sexual dysfunction among men with SCD [7,8]. This pattern is biologically plausible and aligns with long-standing observations that priapism, reported in one-third of our sample, and chronic anemia, hypoxia, and hemolysis progressively damage cavernosal tissue, disrupting nitric oxide signaling and impairing smooth muscle function [13-18,20,21]. These vascular scars coexist with endocrine issues, including hypogonadism and iron overload, which further diminish sexual function and desire [19-21].

From a clinical perspective, the main question is how to identify these men during routine care efficiently. In our study, simplified tools showed strong agreement with IIEF-5: the EHS and a single MMAS-style item demonstrated excellent discrimination (AUC ≥0.82). At the same time, a basic visual analog named “Erectometer” achieved an AUC of 0.90 with high sensitivity and specificity. These findings align with previous evidence that brief instruments can effectively measure the core concept of ED with minimal information loss and reduce respondent burden. In settings with low literacy and in time-constrained clinics, such tools may lower the barrier for systematic screening and enable earlier discussions about medication options, hormone evaluations, and relationship-centered counseling [22-30].

Another relevant aspect is the validity and cultural adaptability of these measures. The EHS and MMAS-derived single-item questions have been adapted and tested in multiple languages, with psychometric properties comparable to the original instruments and offer good responsiveness to clinical use-change, supporting their use across diverse contexts [24,26,27]. The Erectometer, although less widely studied, has been previously associated with both hemodynamic parameters and patient-reported outcomes in ED cohorts [29,30]. Its performance in our sample, with high AUC and balanced sensitivity and specificity, suggests that patients with SCD can intuitively understand a simple visual analog representation of erection quality and may be integrated into routine assessments. Further psychometric work in larger samples will be important to consolidate its role as a screening tool.

Beyond diagnostic accuracy, our results highlight the importance of explicitly addressing sexual health in chronic SCD care. Many men in our cohort are in their third or fourth decade, still in the most productive and relationally active years, yet living with moderate or severe ED. Qualitative and quantitative research has shown that pain, hospitalizations, and uncertainty about future health can lead to anxiety, low self-esteem, and depression in adults with SCD [1-4]. Sexual dysfunction may add to this stress, yet it often remains unaddressed during hematology follow-ups or primary-care visits, either because of time constraints or discomfort in bringing up the topic. Using brief ED screening tools systematically can normalize the conversation, guide targeted referrals (urology, psychology, endocrinology), and help ensure that men with SCD receive care that is both focused on the disease and attentive to their personal needs.

Strengths of this study include standardized measurement procedures, consistent clinical characterization at a reference center, and the use of a validated ED reference instrument (IIEF-5) for comparison. Limitations include the single-center design, modest sample size, and cross-sectional nature, which restrict causal inference and limit generalizability beyond similar settings. The sample may under-represent men with more severe cognitive or communication difficulties, and we did not formally assess depressive symptoms or detailed relationship variables, which could influence perceived sexual function. Future studies should evaluate these instruments in larger, multicenter SCD cohorts, incorporate longitudinal follow-up, and test structured “screen-and-treat” pathways to determine whether routine ED screening improves quality of life and mental health outcomes.

## Conclusions

In conclusion, erectile dysfunction was highly prevalent in this cohort of adult men with sickle cell disease, and simplified screening instruments demonstrated excellent diagnostic performance compared with the IIEF-5.

Brief tools such as the EHS, a single MMAS-style item, and a simple visual analog “Erectometer” accurately captured erectile impairment and may facilitate systematic screening during routine visits, mainly in time-constrained or low-literacy settings.

The modest sample size limits external generalizability, and recruitment from a single specialized center may introduce selection bias. Because the study was cross-sectional, all findings reflect associations rather than causal relationships. Potential psychosocial confounders - such as depressive symptoms, anxiety, and relationship factors - were not formally assessed and may influence perceived sexual function. Non-response may also have affected prevalence estimates and performance measures of simplified tools.

All results should be interpreted as associative rather than causal, consistent with the study design. Confidence intervals and effect sizes complement p-values in describing clinical relevance. The performance of simplified tools suggests they may facilitate structured screening during routine care for men with SCD.

Future multicenter studies with larger samples and longitudinal follow-up are needed to consolidate the role of these instruments, particularly the Erectometer, and to determine whether structured screening pathways can improve sexual health and overall quality of life for men living with SCD.
